# Radix *Salvia miltiorrhiza* for Ankylosing Spondylitis: Determining Potential Inflammatory Molecular Targets and Mechanism Using Network Pharmacology

**DOI:** 10.1155/2022/3816258

**Published:** 2022-09-13

**Authors:** Yanyan Fang, Jian Liu, Ling Xin, Hui Jiang, Jinchen Guo, Xu Li, Fanfan Wang, Mingyu He, Qi Han, Dan Huang

**Affiliations:** ^1^The First Affiliated Hospital of Anhui University of Chinese Medicine, Hefei, Anhui 230038, China; ^2^Key Laboratory of Xin'an Medicine of the Ministry of Education, Anhui University of Chinese Medicine, Hefei, Anhui 230038, China; ^3^Anhui Province Key Laboratory of Modern Chinese Medicine Department of Internal Medicine Application Foundation Research and Development, Hefei, Anhui 230038, China; ^4^Anhui University of Chinese Medicine, Hefei, Anhui 230031, China

## Abstract

Radix *Salvia miltiorrhiza* (RSM) is widely used for the clinical improvement of inflammatory diseases. However, the actions of RSM in the treatment of ankylosing spondylitis (AS) have not been fully explored. Therefore, this study was designed to use retrospective clinical data mining approach to understand the effects of RSM on AS-related immuno-inflammatory processes, use network pharmacology to predict therapeutic targets of RSM, and to further investigate the pharmacological molecular mechanism *in vitro*. RSM treatment has a long-term correlation with the improvement of AS-related immuno-inflammatory indicators through computational models. We established protein-protein interaction networks, conducted KEGG analysis to enrich significant TNF pathways, and finally obtained three core targets of RSM in the treatment of AS, namely, prostaglandin endoperoxide synthase 2 (PTGS2), interleukin-6 (IL-6), and tumor necrosis factor-alpha (TNF-alpha). Screening of RSM active ingredients with node degree greater than 20 yielded cryptotanshinone and tanshinone IIA, and previous studies have reported their anti-inflammatory effects. *In vitro*, both cryptotanshinone and tanshinone IIA significantly inhibited the expressions of PTGS2, IL-6, and TNF-*α* in peripheral blood mononuclear cells in AS patients. In conclusion, cryptotanshinone and tanshinone IIA, which are the active components of RSM, may inhibit the activation of TNF signaling pathway in AS patients by downregulating the expression of PTGS2, IL-6, and TNF-*α*. These findings illustrate that RSM may be a promising therapeutic candidate for AS, but further validation is required.

## 1. Introduction

Ankylosing spondylitis (AS) is a chronic inflammatory disease characterized by inflammatory back pain and asymmetric peripheral joint synovitis [1]. The global prevalence of AS is between 0.07% and 0.32% [2], and the incidence within the male population is significantly higher than that within the female population [3]. Moreover, the age of onset of AS is relatively low, usually between 26 and 45 years [3]. If not diagnosed and treated in time, AS may cause joint damage and disability, which may seriously affect patient's health and quality of life [4–6]. According to existing research, the pathogenesis of AS is related to many factors; however, the specific etiology is unclear [7]. At present, the most common clinical treatments for AS focus on relieving symptomatic pain; however, the long-term effects of these treatments are not ideal and often lead to side effects [8]. Therefore, the development of new AS treatment strategies is essential.

Radix *Salvia miltiorrhiza* (RSM), also known as Danshen in China, is the root and rhizome of *Salvia miltiorrhiza*, a plant of the Lamiaceae family. Danshen has been widely used to treat various diseases, including those of the cardiovascular and immune systems [9, 10]. In clinical studies, RSM has been proven to have anti-inflammatory effects because of the rich phenolic acids and tanshinones in the stems and leaves [11]. However, few studies have evaluated the mechanisms of action of RSM in the treatment of AS, and further research is needed in this regard.

Data mining technology explores potentially valuable knowledge derived from a large amount of data and has been widely used in the medical domain [12]. Retrospective data mining research can generate meaningful insights from clinical datasets. Network pharmacology is a novel and convenient method of discovering bioactive components, predicting drug action targets, and analyzing drug action mechanisms using data from public repositories [13]. The ability to evaluate the multichannel regulation of signaling pathways is an added advantage of network pharmacology, making it particularly suitable for understanding the mechanisms of action of traditional Chinese medicines with varying chemical compositions and molecular targets [14].

Considering the complexity of traditional Chinese medicines and their functions, network pharmacology provides a convenient way of determining molecular targets. Therefore, in this study, we aimed to explore the molecular targets of RSM in the treatment of AS using network pharmacology. Using this approach, we sought to predict the possible mechanisms of action for the treatment of AS based on the identified targets and signal transduction pathways, verified these targets using molecular docking, and conducted *in vitro* experiments to verify the therapeutic effects of the active ingredients of RSM for AS.

## 2. Materials and Methods

### 2.1. Acquisition of Clinical Data and Analysis of Association Rules

Data regarding immuno-inflammation indices and RSM usage between July 2009 and June 2021 were obtained from the records of 2074 patients with AS from the Department of Rheumatology and Immunology, Anhui Provincial Hospital of Traditional Chinese Medicine. The immuno-inflammation indices included the erythrocyte sedimentation rate (ESR) and C-reactive protein (CRP), immunoglobulin A (IgA), immunoglobulin M (IgM), immunoglobulin G (IgG), complement component 3 (C3), and complement component 4 (C4) levels.

The *apriori* algorithm module in the IBM SPSS Modeler 18.0 software (IBM Corp., Armonk, N.Y., USA) was adopted to analyze the association between RSM and immuno-inflammation indicators [15].

### 2.2. Random Walking

The Oracle Developer Suite 10 g (Oracle Corp., Austin, Texas, USA) was used to evaluate the random walking model of the immuno-inflammation indices and to observe drug compatibility-based improvements in laboratory indices [16].

### 2.3. Identification of the Effective Chemical Components of RSM and Corresponding Molecular Targets

We used the Traditional Chinese Medicine Systems Pharmacology (TCMSP, http://tcmspw.com/tcms) [17] database to screen chemical components that meet oral bioavailability ≥30% and drug-likeness ≥0.18 as the effective ingredients of RSM. Further, we used the TCMSP database to screen overall RSM effectiveness. The molecular target of the RSM component was entered into the UniProt database (https://www.uniprot.org). We limited the species to “homo sapiens” and corrected the gene names of the target proteins.

### 2.4. Collection of AS-Related Genes

We searched the GeneCards database (https://www.genecards.org), Online Mendelian Inheritance in Man (OMIM, https://omim.org/), Pharmacogenetics and Pharmacogenomics Knowledge Base (PharmGKB, https://www.pharmgkb.org/), and the Therapeutic Target Database (TTD; http://db.idrblab.net/ttd/) using “ankylosing spondylitis” as the key word. We removed the duplicate targets to obtain the unique AS target gene.

### 2.5. Determination of Potential Targets for RSM in the Treatment of AS

We used the Venny 2.1.0 database (https://bioinfogp.cnb.csic.es/tools/venny/) to develop a Venn diagram, obtain the drug-disease interactions, and use these interactions to evaluate potential targets for RSM in the treatment of AS.

### 2.6. Construction of the Protein–Protein Interaction Network and Screening of Key Targets

In this study, we imported the potential target genes of RSM for the treatment of AS into the STRING database (https://string-db.org/), limited the species to “homo sapiens” with a minimum confidence score of ≥0.400, saved the results in TSV format, and imported them into the Cytoscape 3.8.0 software (http://www.cytoscape.org/) to draw the target protein interaction network. We then used the Bisogenet plug-in [18] to construct a protein–protein interaction (PPI) network of key targets. Using average nodal degree as a screening parameter, MCC identified the top five results as the key targets of RSM.

### 2.7. Annotation of Target Pathway Enrichment Analysis

Bioinformatics (http://www.bioinformatics.com.cn/) was used to obtain the targets of RSM for Kyoto Encyclopedia of Genes and Genomes (KEGG) pathway enrichment analysis. The KEGG bubble map for the first 10 significantly enriched disease-related pathways were selected.

### 2.8. Molecular Docking of Active Ingredients with Key Target Genes

We downloaded the molecular structures of the key target proteins from the Protein Data Bank (PDB) database and used AutoDock1.5.6 software to remove water molecules, hydrogenate the molecular and active ingredient structures, and perform semiflexible binding and molecular docking verification. A genetic algorithm was used for molecular docking calculations. Each docking was performed 50 times, and the maximum operation was 300. Default values were used as docking parameters. Binding activities between the ligand and the receptor were indicated if the binding energy was <0. If the binding energy was <-5 kJ/mol, the two were considered well connected. Finally, PyMOL was used to visualize the image of the site on the docking molecule with the lowest binding energy.

### 2.9. Sample Collection and Culture

Nine patients with AS from the Department of Rheumatology and Immunology, Anhui Provincial Hospital of Traditional Chinese Medicine, were randomly divided into a nontreatment group (NT), AS+cryptotanshinone group (AS+cryptotanshinone), and AS+tanshinone IIA group (AS+tanshinone IIA). Three healthy subjects were included in the healthy control group (HC). The peripheral blood mononuclear cells (PBMCs) of each group were extracted and seeded in 6-well cell culture plates. The culture plates were supplemented with Roswell Park Memorial Institute-1640 (HyClone, USA) containing 10% fetal bovine serum (Sigma-Aldrich) and 1% streptomycin and penicillin (Beyotime, Shanghai, China) after adjusting the cell density to 1 × 10^6^/mL and incubated for 24 hours. Both cryptotanshinone and tanshinone IIA were purchased from MedChemExpress (MCE, Shanghai, China), and the both drugs dose used were those reported by the MCE official website for *in vitro* experiments [19, 20]. The final working solution of cryptotanshinone with a concentration of 7 *μ*mol/L was added to the AS+cryptotanshinone group, and incubated in an incubator for 24 hours. The final working solution of tanshinone IIA with a concentration of 145 *μ*mol/L was added to the AS+tanshinone IIA group, and incubated in an incubator for 24 hours. All PBMCs were maintained in incubator at 37°C and 5% CO_2_.

### 2.10. Western Blotting

Peripheral blood mononuclear cells were lysed in radioimmunoprecipitation assay (RIPA) buffer (Biosharp, China) containing protease and phosphatase inhibitors (Biosharp, China) to extract total protein. The primary antibodies used in this study included anti-*β*-actin (1 : 1000, TA-09; Zs-BIO, China), anti-COX2 (1 : 1000, AF7003; Affinity, China), anti-IL-6 (1 : 1000, DF6087; Affinity, China), and anti-TNF-*α* (1: 1000, AF7014; Affinity, China). The secondary antibodies included goat anti-rabbit IgG (ZB-2301; Zs-BIO, China), anti-*β*-actin (1: 10000, TA-09; Zs-BIO, China), anti-COX2 (1: 5000, AF7003; Affinity, China), anti-IL-6 (1: 5000, DF6087; Affinity, China), and anti-TNF-*α* (1: 5000, AF7014; Affinity, China).

### 2.11. Statistical Analysis

GraphPad Prism 8.2 software was used to analyze the data. Data of immuno-inflammation indices were expressed as the median (Q1, Q3). Continuous variables were analyzed using the Wilcoxon signed-rank test, since the immuno-inflammation indices were not normally distributed. Western blotting results were expressed as the mean ± SD (standard deviation), and were analyzed using Student's *t* test. A *p* value <0.05 was considered statistically significant.

## 3. Results

### 3.1. Changes to Immuno-Inflammatory Indices in Patients with AS

The clinical indices were lower after RSM treatment than before treatment shown in Table 1 (clinical indices: ESR, CRP, IgA, IgG, C3, and C4; *p* < 0.01).

### 3.2. Association between RSM and Immuno-Inflammation Indices

Setting the support degree >20%, the confidence degree >100%, and lift >1 revealed a strong association between RSM treatment and the following clinical indices: ESR, CRP, IgA, IgG, C3, and C4 (Table 2).

### 3.3. Random Walking

**Figure 1:**
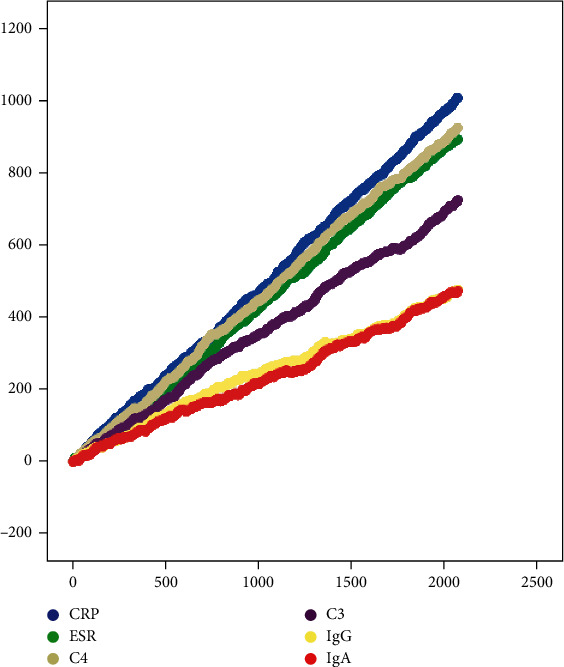
Random walking model of immuno-inflammatory indices in AS patients.

The results of the random walking model identified an improved long-term correlation between RSM treatment and ESR, CRP, IgA, IgG, C3, and C4 levels in patients with AS (Figure 1).

### 3.4. RSM-Disease-Active Ingredients of RSM-Target Network

**Figure 2:**
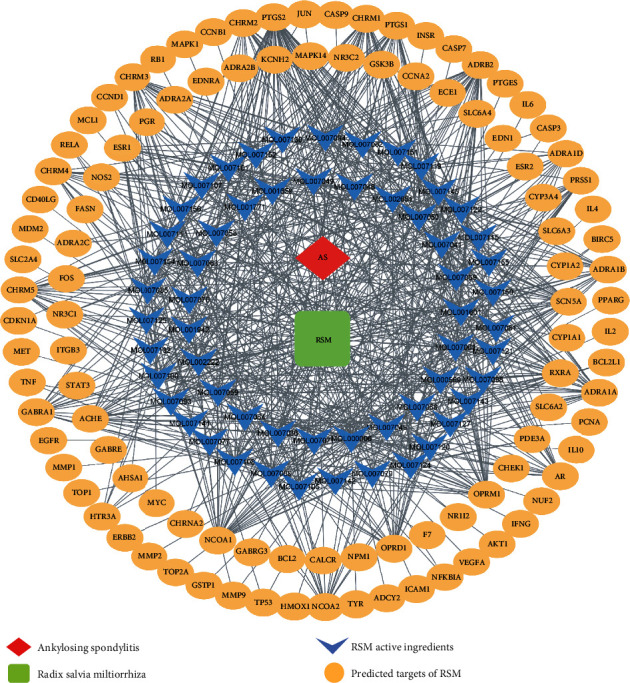
RSM-diseases-active ingredients of RSM-target network. RSM: Radix *Salvia miltiorrhiza.*

According to the parameters of OB ≥30% and DL ≥0.18, 65 active ingredients of RSM and 104 target proteins were identified using TCMSP screening. A total of 2468 AS-related targets were screened using the GeneCards, OMIM, PharmGKB, and TTD databases. The drug-disease-pharmaceutical active ingredient-target network was visualized using Cytoscape shown in Figure 2.

### 3.5. Determining the Targets of RSM in the Treatment of AS

**Figure 3:**
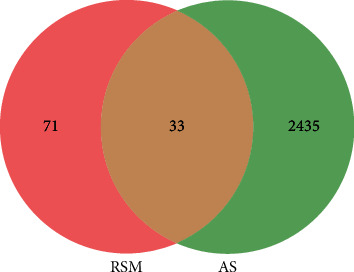
Venn diagram of Radix *Salvia miltiorrhiza* and disease-related targets. RSM (Radix *Salvia miltiorrhiza)* represents Radix *Salvia miltiorrhiza* targets, and AS (ankylosing spondylitis) represents disease-related targets.

Venn diagram was constructed to identify RSM- and AS-related targets. The analysis returned 33 common targets, which was shown in Figure 3.

### 3.6. PPI Network Construction and Key Target Screening

**Figure 4:**
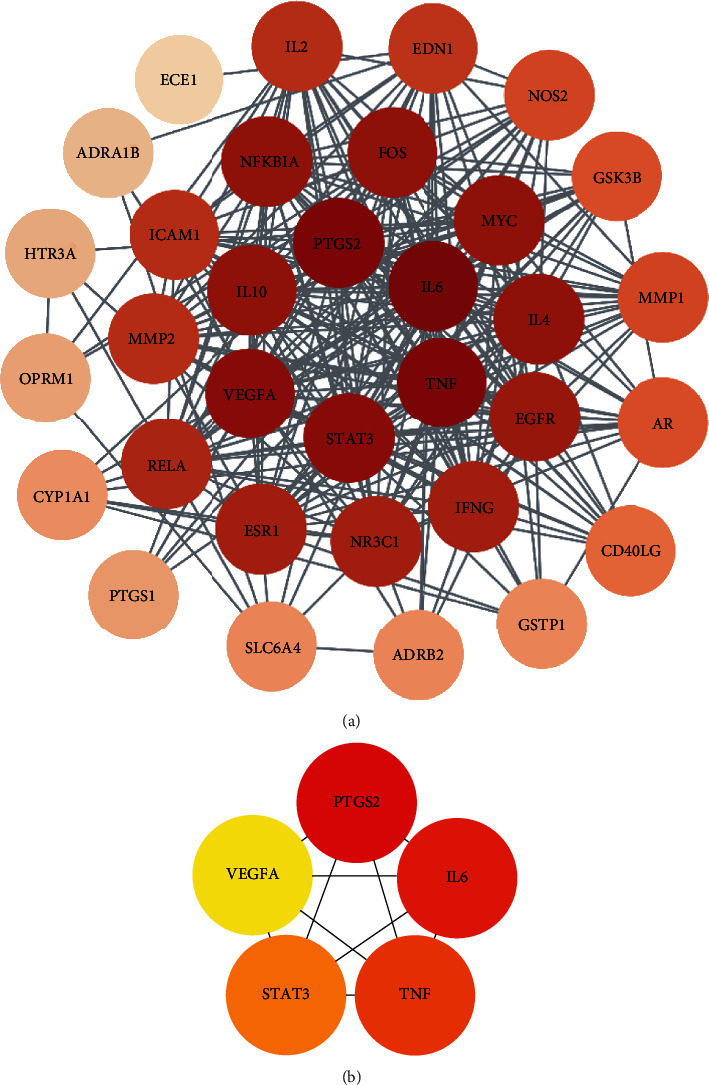
(a) The PPI network of Radix *Salvia miltiorrhiza* in the treatment of AS and (b) the identified core targets. The darker the color, the higher was the degree of interaction.

The common targets were imported into Cytoscape to construct a drug target PPI interaction network (Figure 4(a)). The darker the color, the higher was the degree of interaction between the effective components of RSM and the AS-related molecular target. To further screen for the core targets of the effective components of RSM, we used MCC to identify the five highest scoring candidates, namely, prostaglandin-endoperoxide synthase 2 (PTGS2), interleukin-6 (IL-6), tumor necrosis factor (TNF), signal transducer and activator of transcription 3 (STAT3), and vascular endothelial growth factor A (VEGFA; Figure 4(b)).

### 3.7. KEGG Pathway Enrichment Analysis

**Figure 5:**
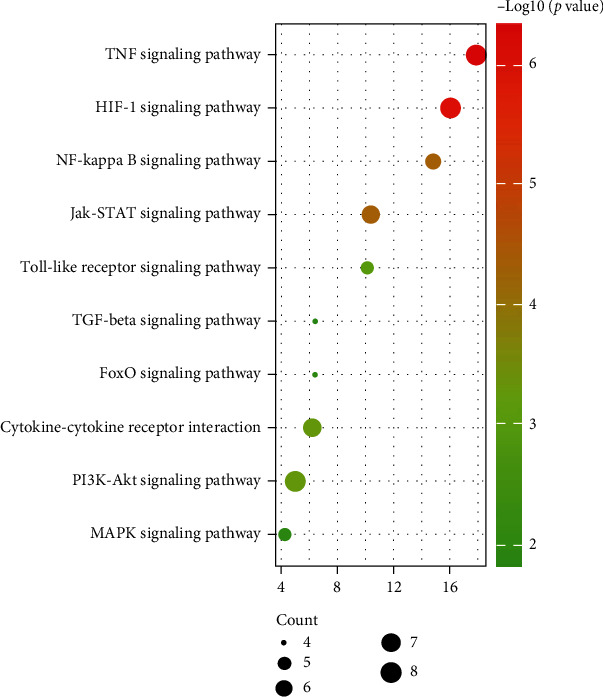
The results of KEGG pathway enrichment analysis for the activities of RSM in patients with AS. KEGG: Kyoto Encyclopedia of Genes and Genomes; RSM: Radix *Salvia miltiorrhiza*; AS: ankylosing spondylitis.

KEGG pathway enrichment analysis identified the following pathways as the 10 pathways with the highest degree of common target enrichment: TNF, HIF-1, NF-*κ*B, Jak-STAT, Toll-like receptor, TGF-beta, FoxO, cytokine–cytokine receptor interaction, PI3K-Akt, and MAPK signaling pathway (Figure 5).

### 3.8. Molecular Docking Verification

**Figure 6:**
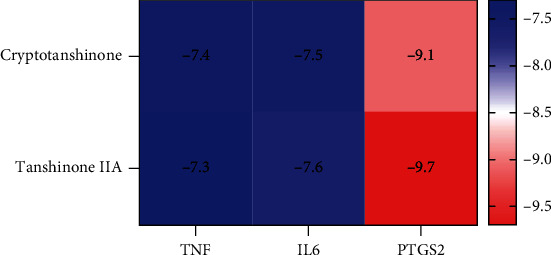
The binding energies of the chemically active RSM ingredients and the key target proteins. RSM: Radix *Salvia miltiorrhiza.*

**Figure 7:**
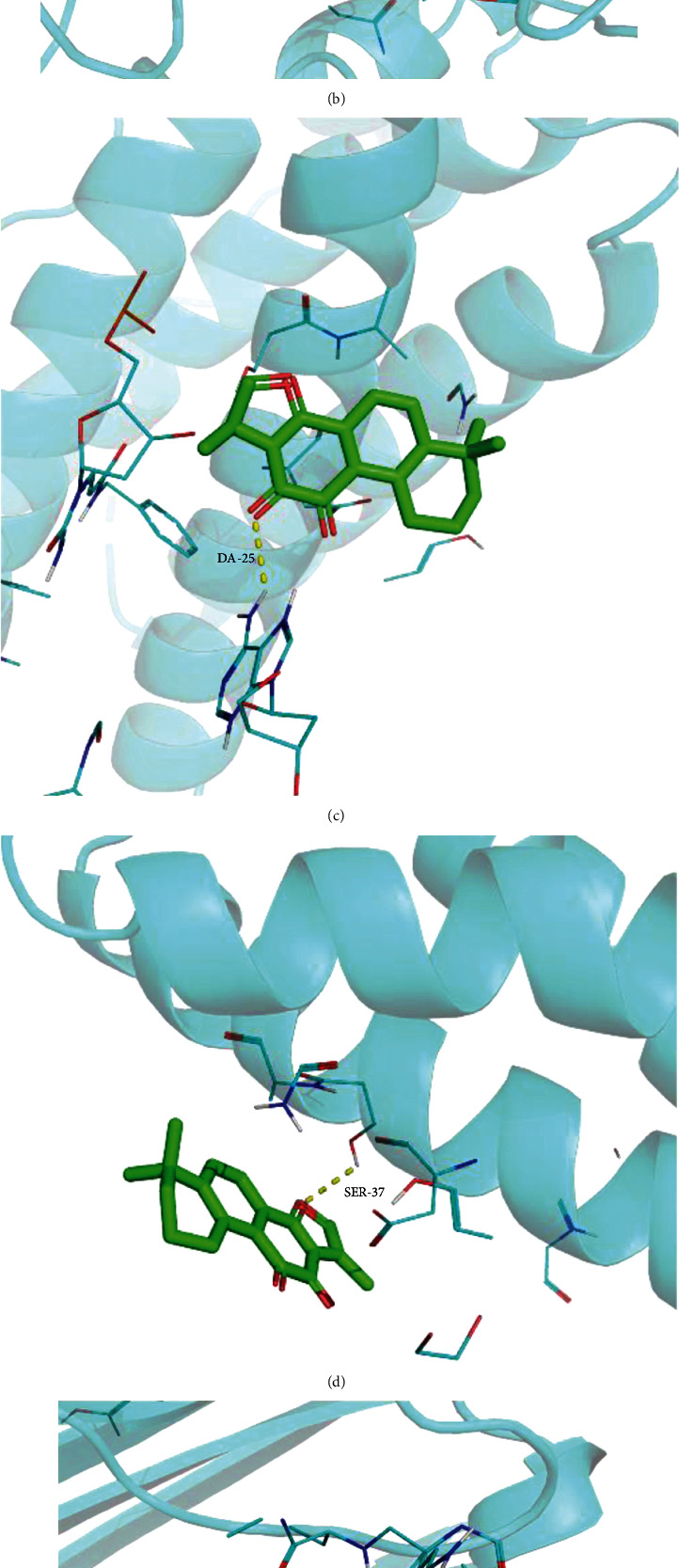
The docking patterns of the inflammatory targets of the network and the lowest components of their binding energies. (a) The docking diagram of cryptotanshinone (MOL007088) and PTGS2 (5f19). (b) The docking diagram of tanshinone IIA (MOL007154) and PTGS2 (5f19). (c) The docking diagram of cryptotanshinone (MOL007088) and IL-6 (4ni7). (d) The docking diagram of tanshinone IIA (MOL007154) and IL-6 (4ni7). (e) The docking diagram of cryptotanshinone (MOL007088) and TNF (5uui). (f) The docking diagram of tanshinone IIA (MOL007154) and TNF (5uui).

The active components of RSM (i.e., those with a node degree greater than 20) were screened, and previous studies were reviewed to positively identify cryptotanshinone and tanshinone IIA as the components that may be most effective for the treatment of AS. *PTGS2, IL-6,* and *TNF* are shared by the TNF signaling pathway (the most enriched pathway) and were the key target genes identified using the MCC algorithm. Therefore, the active ingredients cryptotanshinone and tanshinone IIA were selected for molecular docking with the shared target genes *PTGS2*, *IL-6*, and *TNF*. The results showed that binding energies between effective components and PTGS2, IL-6, and TNF were less than -5 kJ·mol^−1^, indicating that all have good docking activity (Figure 6) and visualization processing (Figure 7).

### 3.9. Mechanism of Cryptotanshinone and Tanshinone IIA on PTGS2, IL-6, and TNF-*α* Protein Expression

**Figure 8:**
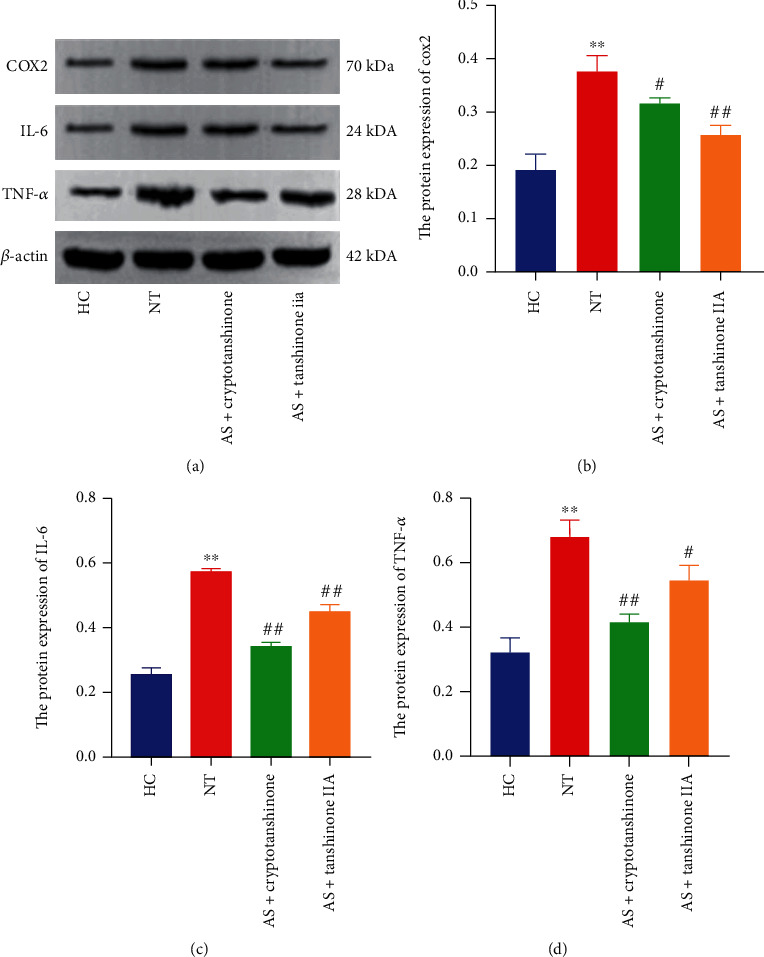
Effects of cryptotanshinone and tanshinone IIA on PTGS2, IL-6, and TNF-*α* protein expression. (a) Visual protein expression levels in peripheral blood mononuclear cells from the healthy control group (HC), nontreatment group (NT), AS+cryptotanshinone group (AS+cryptotanshinone), and AS+tanshinone IIA group (AS+tanshinone IIA). (b) Quantitative expression of PTGS2 (COX2) in each group. (c) Quantitative expression of IL-6 in each group. (d) Quantitative expression of TNF-*α* in each group. Data are represented as means ± SD. ^∗^*p* < 0.05 and ^∗∗^*p* < 0.01, compared with the normal control group. ^#^*p* < 0.05 and ^##^*p* < 0.01, compared with the NT group. The experiments were independently repeated at least three times.

The expression of PTGS2, IL-6, and TNF-*α* proteins in the NT group was significantly higher than that of these proteins in the HC group (*p*_PTGS2_ = 0.0016, *p*_IL−6_ < 0.0001, *p*_TNF−*α*_ = 0.0008). The protein expression levels of the AS+cryptotanshinone and AS+tanshinone IIA groups were significantly lower than those of the NT group (*p*_PTGS2_ = 0.0316, *p*_PTGS2_ = 0.0040, *p*_IL−6_ < 0.0001, *p*_IL−6_ = 0.0007, *p*_TNF−*α*_ = 0.0013, and *p*_TNF−*α*_ = 0.0264), indicating that the active ingredients of RSM can significantly reduce the expression of PTGS2, IL-6, and TNF-*α* (Figure 8).

## 4. Discussion

AS is a common autoimmune disease characterized by inflammation and aberrant osteogenesis, but the pathophysiology of AS has not been fully elucidated. Recently, research has shown that the inflammatory state is closely related to a variety of chronic rheumatic diseases, such as rheumatoid arthritis and psoriatic arthritis [21]. Anti-inflammatory treatment is becoming increasingly valued by researchers and clinicians. However, based on the progression of inflammation-mediated diseases, the long-term use of steroidal or nonsteroidal anti-inflammatory drugs is not advisable. Therefore, there is a need to find safe and effective anti-AS drugs. Cryptotanshinone, an active ingredient of RSM, has anti-inflammatory and immunomodulatory effects [22] and is widely used in the treatment of inflammatory-mediated diseases. Although studies have deciphered the active ingredients and molecular mechanisms responsible for the anti-inflammatory effects of RSM [23], few studies have explored the underlying mechanisms of RSM for the treatment of AS using network pharmacology. In this study, we used network pharmacology and molecular docking methods to predict the molecular mechanisms of action of RSM in the treatment of AS.

PTGS2, also known as cyclooxygenase-2 (COX2), is a key, rate-limiting enzyme that catalyzes the synthesis of prostaglandins from arachidonic acid [24]. COX2 converts arachidonic acid into prostaglandin E2 (PGE2) during inflammation and amplifies the Th17-mediated autoimmune process through the COX2-PGE2-EP2 axis [25]. Therefore, PTGS2 may be a potential target for therapeutic intervention in AS. In our findings, PTGS2 ranked above all candidate targets using the MCC algorithm. Based on the information in the public database, we predicted that RSM affects PTGS2 expression, and their interaction is supported by the molecular docking findings. In our *in vitro* verification experiments, the same result was obtained. Both tanshinone IIA and cryptotanshinone were shown to reduce the expression of PTGS2. Our results indicate that PTGS2 is the key target of RSM in the treatment of AS. These results are consistent with clinical studies, and drugs that block COX2 activity are recommended as first-line clinical treatments for patients with AS [26].

According to previous reports, high levels of the inflammatory cytokines IL-6 and TNF are closely related to the severity of AS [27]. Interestingly, tanshinone IIA (a diterpene isolated from the root of RSM) has a notable inhibitory effect on the production of nitric oxide, IL-1*β*, IL-6, and TNF-*α* [28]. Zhang et al. [8] found that the RSM water extract, PF2401-SF (enriched with tanshinone I, tanshinone IIA, and cryptotanshinone), significantly reduced carrageenan- or dextran-induced acute arthritis in rats. These findings corroborate that RSM, which acts as an anti-inflammatory herb, can inhibit inflammation by reducing the levels of IL-6 and TNF-*α*.

According to our findings and those of previous studies, STAT3 and VEGFA may also be key targets for AS treatment. Inhibition of STAT3 phosphorylation is a promising treatment for countering AS inflammation and osteogenesis [29]. Angiogenesis is closely related to the pathogenesis of chronic inflammatory diseases, and inflammation can promote the upregulation of VEGF in rheumatic diseases [30]. The disease state of AS appears to be associated with elevated levels of VEGF in peripheral blood [31]. Therefore, VEGF may be a key participant in the pathogenesis of AS and a target for therapeutic intervention.

KEGG analysis showed that the TNF signaling pathway is the most enriched pathway in patients with AS treated with RSM. Consistent with our results, cryptotanshinone can inhibit the production of TNF-*α* induced by IL-1*β* [32]. Similarly, cryptotanshinone can reduce the severity of collagen-induced arthritis by inhibiting the production of TNF-*α* and downregulating the production and activity of matrix metalloproteinase-9 [33]. In addition to PTGS2 and TNF, we also docked IL-6, the downstream target of the TNF signaling pathway, with the active ingredients of RSM. These results show that cryptotanshinone and tanshinone IIA form hydrogen bonds with the predicted targets PTGS2, IL-6, and TNF, indicating that PTGS2, IL-6, and TNF may be candidate targets for the treatment of AS by RSM.

*In vitro* cell experiments further verified the effects of tanshinone IIA and cryptotanshinone on PTGS2, IL-6, and TNF-*α* expression levels, and the findings are consistent with our predicted results. Both tanshinone IIA and cryptotanshinone can significantly inhibit the expression levels of PTGS2, IL-6, and TNF-*α*. Although RSM is clinically effective, its single therapeutic effect is relatively weak, and the sample size of our *in vitro* verification experiments is small. Thus, the sample size should be increased in future studies to improve the reliability of the experimental findings.

In conclusion, RSM has a long-term correlation with the improvement of clinical immuno-inflammatory indicators in AS. The therapeutic mechanisms of action may be that the active ingredients, cryptotanshinone and tanshinone IIA, inhibit the activation of the TNF-*α* signaling pathway among other inflammatory responses induced in AS, thereby inhibiting the expression of PTGS2, IL-6, and TNF. The multitarget drug, RSM, may be a promising therapeutic candidate for AS, but further experimentation with larger sample sizes is needed.

## Figures and Tables

**Table 1:** Changes in immune-inflammatory indices after treatment with RSM (*n* = 2074).

	Before treatment	After treatment	*p* value	*Z*
ESR [median (Q1, Q3), mm/h]	28.00 (14.00, 49.00)	21.00 (12.00, 37.00)	≤0.001	-16.986
CRP [median (Q1, Q3), mg/L]	18.215 (5.44, 38.70)	8.025 (1.90, 21.2125)	≤0.001	-21.266
IgA [median (Q1, Q3), g/L]	2.59 (1.87, 3.51)	2.44 (1.81, 3.305)	≤0.001	-10.594
IgM [median (Q1, Q3), g/L]	1.13 (0.825, 1.48)	1.13 (0.84, 1.50)	0.359	-.916
IgG [median (Q1, Q3), g/L]	13.07 (10.68, 15.80)	12.51 (10.30, 15.00)	≤0.001	-9.013
C3 [median (Q1, Q3), g/L]	120.50 (81.20, 139.50)	111.50 (75.95, 129.80)	≤0.001	-11.391
C4 [median (Q1, Q3), g/L]	28.50 (16.25, 35.80)	24.60 (14.15, 31.05)	≤0.001	-16.504

Note: ESR: Erythrocyte sedimentation rate; CRP: C-reactive protein; IgA: immunoglobulin A; IgM: immunoglobulin M; IgG: immunoglobulin G; C3: complement component 3; C4: complement component 4. *p* value is based on the comparison between levels before and after treatment. *Z* is the standardized test statistics before and after treatment.

**Table 2:** Association between RSM and immuno-inflammation indices.

Drug	Clinical indices	Support (%)	Confidence (%)	Lift
RSM	CPR	25.422	100	3.934
RSM	ESR	32.176	100	3.108
RSM	IgA	61.891	100	1.616
RSM	IgG	61.891	100	1.616
RSM	C3	61.891	100	1.616
RSM	C4	61.891	100	1.616

RSM: Radix *Salvia miltiorrhiza*; ESR: erythrocyte sedimentation rate; CRP: C-reactive protein; IgA: immunoglobulin A; IgG: immunoglobulin G; C3: complement component 3; C4: complement component 4.

## Data Availability

The data used in this study are available from the corresponding author at reasonable request.
