# Correction to “Radix *Salvia miltiorrhiza* for Ankylosing Spondylitis: Determining Potential Inflammatory Molecular Targets and Mechanism Using Network Pharmacology”

**DOI:** 10.1155/bmri/9818327

**Published:** 2025-10-16

Y. Fang, J. Liu, L. Xin, et al., “Radix *Salvia miltiorrhiza* for Ankylosing Spondylitis: Determining Potential Inflammatory Molecular Targets and Mechanism Using Network Pharmacology,” *BioMed Research International* 2022 (2022): 3816258, https://doi.org/10.1155/2022/3816258

In the article, an error was introduced during the production process, where in Figure 8a, the bands for COX2 were duplicated with the IL‐6 bands. The correct Figure 8 is shown in the following:

Figure 8Effects of cryptotanshinone and tanshinone IIA on PTGS2, IL‐6, and TNF‐*α* protein expression. (a) Visual protein expression levels in peripheral blood mononuclear cells from the healthy control group (HC), nontreatment group (NT), AS+cryptotanshinone group (AS+cryptotanshinone), and AS+tanshinone IIA group (AS+tanshinone IIA). (b) Quantitative expression of PTGS2 (COX2) in each group. (c) Quantitative expression of IL‐6 in each group. (d) Quantitative expression of TNF‐*α* in each group. Data are represented as means ± SD.  ^∗^ *p* < 0.05 and  ^∗∗^ *p* < 0.01, compared with the normal control group. ^#^ *p* < 0.05 and ^##^ *p* < 0.01, compared with the NT group. The experiments were independently repeated at least three times.

**Figure:**
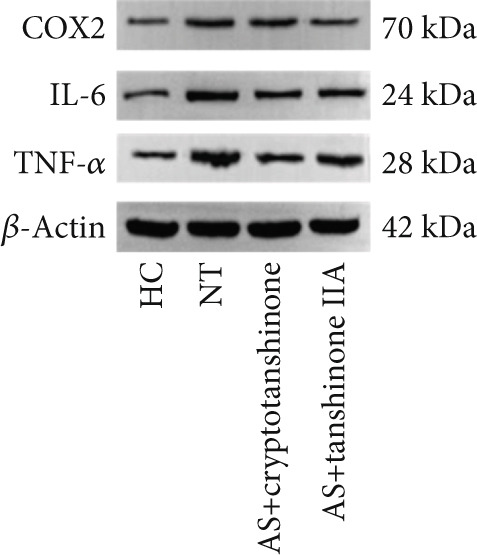
(a)

**Figure:**
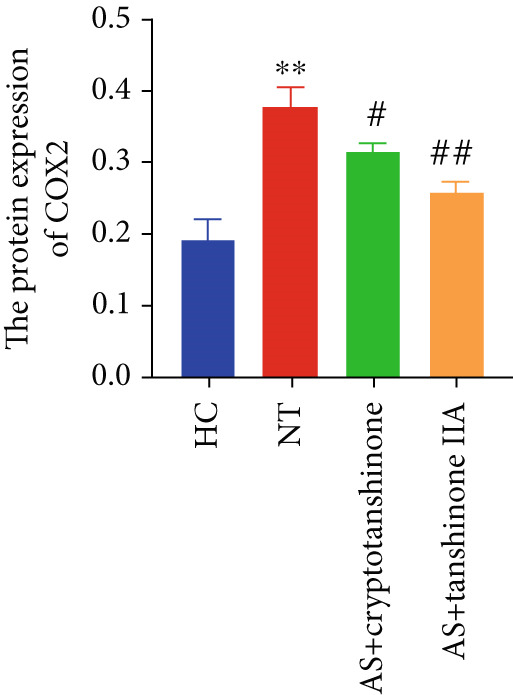
(b)

**Figure:**
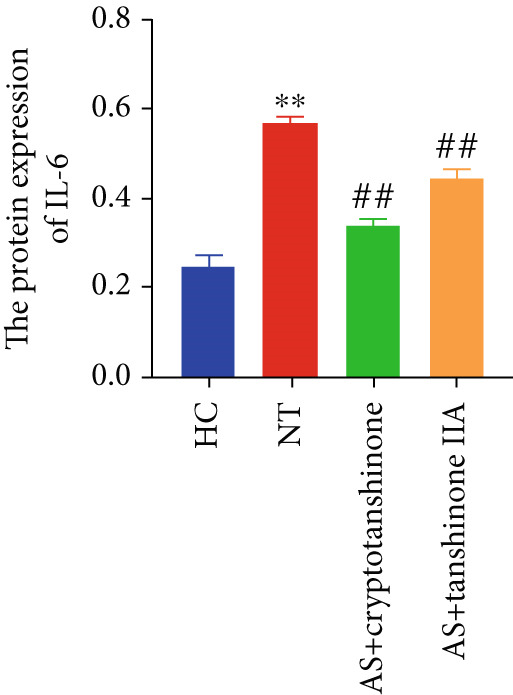
(c)

**Figure:**
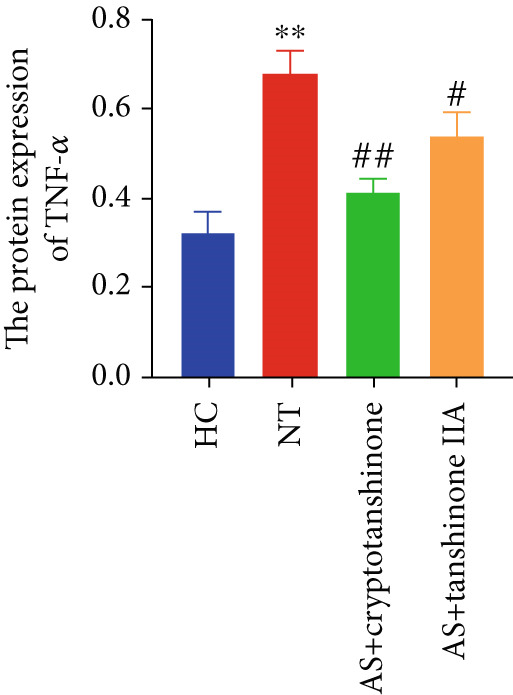
(d)

We apologize for this error.

